# Federated Analysis With Differential Privacy in Oncology Research: Longitudinal Observational Study Across Hospital Data Warehouses

**DOI:** 10.2196/59685

**Published:** 2025-07-31

**Authors:** Théo Ryffel, Perrine Créquit, Maëlle Baillet, Jason Paumier, Yasmine Marfoq, Olivier Girardot, Thierry Chanet, Ronan Sy, Louise Bayssat, Julien Mazières, Vincent Vuiblet, Julien Ancel, Maxime Dewolf, François Margraff, Camille Bachot, Jacek Chmiel

**Affiliations:** 1 Arkhn, 9, rue d'Alexandrie, Paris, 75002, France; 2 Hôpital Foch, Suresnes, France; 3 Centre Hospitalier Universitaire de Toulouse, Service de Pneumologie, Toulouse, France; 4 INSERM, Paris, France; 5 Centre de Recherches en Cancérologie Oncopole, Toulouse, France; 6 Centre Hospitalier Universitaire de Reims, Service de Pneumologie, Reims, France; 7 Roche SAS, Boulogne, France

**Keywords:** federated analysis, differential privacy, real-world oncology study, non-small cell lung cancer, COVID-19, lung cancer, cancer, privacy, oncology, oncology study, cell lung cancer, oncology data

## Abstract

**Background:**

Federated analytics in health care allows researchers to perform statistical queries on remote datasets without access to the raw data. This method arose from the need to perform statistical analysis on larger datasets collected at multiple health care centers while avoiding regulatory, governance, and privacy issues that might arise if raw data were collected at a central location outside the health care centers. Despite some pioneering work, federated analytics is still not widely used on real-world data, and to our knowledge, no real-world study has yet combined it with other privacy-enhancing techniques such as differential privacy (DP).

**Objective:**

The first objective of this study was to deploy a federated architecture in a real-world setting. The oncology study used for this deployment compared the medical health care management of patients with metastatic non–small cell lung cancer before and after the first wave of COVID-19 pandemic. The second goal was to test DP in this real-world scenario to assess its practicality and use as a privacy-enhancing technology.

**Methods:**

A federated architecture platform was set up in the Toulouse, Reims, and Foch centers. After harmonization of the data in each center, statistical analyses were performed using DataSHIELD (Data aggregation through anonymous summary-statistics from harmonized individual-level databases), a federated analysis R library, and a new open-source DP DataSHIELD package was implemented (dsPrivacy).

**Results:**

A total of 50 patients were enrolled in the Toulouse and Reims centers and 49 in the Foch center. We have shown that DataSHIELD is a practical tool to efficiently conduct our study across all 3 centers without exposing data on a central node, once a sufficient setup has been established to configure a secure network between hospitals. All planned aggregated results were successfully generated. We also observed that DP can be implemented in practice with promising trade-offs between privacy and accuracy, and we built a library that will prove useful for future work.

**Conclusions:**

The federated architecture platform made it possible to run a multicenter study on real-world oncology data while ensuring strong privacy guarantees using differential privacy.

## Introduction

Federated analytics (FA) [1] allow researchers to perform statistical queries on remote datasets without accessing raw data. This method has emerged from the need of conducting statistical analyses on larger datasets originating from multiple health care centers, while avoiding regulation, governance, and privacy related issues that could occur if the raw data were gathered in a central location external to the health care centers.

DataSHIELD (Data aggregation through anonymous summary-statistics from harmonized individual-level databases) [2] is one of the pioneering open-source software solutions for bioscience collaboration using federated analysis. It has been used in several projects including the EU Child Cohort Network [3] and German MIRACUM consortium [4]. However, even if it has been identified as a next step in some oncology studies [5], to the best of our knowledge it has not been used on real-world oncology data. One possible reason for this can be that data harmonization is actually the sticking point, since it proves to be challenging on real-world data when not already done ahead of time.

While FA ensures that sensitive data are never directly exposed, results from statistical queries can still leak some information from individuals. Indeed, several privacy attacks [6,7] have been proposed to exploit common statistical analysis results and disclose private information. To mitigate these attacks, differential privacy (DP) can be used in combination with FA to provide stronger privacy guarantees. DP [8,9] is a method for computing statistical analyses on a sensitive dataset in such a way that the results do not compromise the privacy of the initial raw data. It proves particularly appropriate for studies on patient health data which are highly sensitive, but as far as we know, no real-world data study have already combined FA and privacy-enhancing techniques such as DP. Our study focuses on a scenario with 3 hospital nodes, consistent with typical real-world deployments in health care, where the number of participating centers is usually small (on the order of a few nodes). Scalability and performance metrics (eg, query response times and network latency) were therefore not the primary focus of this pilot.

The goal of this study, called Distributed Analytics for Research in Hospitals (DARAH), is to evaluate the operational deployment of a federated architecture in the context of a real-world oncology study. Another objective is to analyze the impact of DP in a federated analysis on the meaningfulness of the study results.

## Methods

### Use of DataSHIELD

At the time of starting the project, we evaluated multiple FA frameworks and chose DataSHIELD due to its comprehensive privacy controls, stable community support, and mature set of statistical functions. DataSHIELD’s unique strength lies in its local, decentralized authorization mechanism and built-in privacy disclosure checks, which prevent analysts from executing arbitrary code on remote servers. The “default privacy disclosure mode” enforces a baseline set of privacy checks (eg, thresholds on minimum cell counts) that align with real-world clinical data governance needs. While other tools like NVidia FLARE or custom Python/R frameworks exist, they often lack these out-of-the-box privacy safeguards, making DataSHIELD a practical choice for our use case. We maintained the default privacy settings throughout the study to ensure a fair demonstration of DataSHIELD’s capabilities without additional configuration.

### Use of Differential Privacy

DP provides a measurement of the privacy risk associated with publishing each particular result on a sensitive dataset, by measuring the maximum leakage that each result can cause about the individuals’ data. In practice, it often works through the addition of a controlled amount of statistical noise to obscure the contributions to the result from each individual in the dataset.

Several open-source libraries exist that implement DP, most of which are written in Python or expose a Python interface. Popular ones include Google’s DP library [10], PyDP [11] from the OpenMined community and OpenDP [12]. In R, the main open-source library is the diffpriv library [13] that implements some DP mechanisms but not a real suite of functions ready to use, and which is not actively supported with last contributions going back to 2017. Furthermore, none of these tools were directly compatible with the DataSHIELD framework, which requires a specific interface in R. As a result, a new open-source a DP R package (R Core Team) for DataSHIELD has been implemented: dsPrivacy. It is available in the Arkhn GitHub repository.

dsPrivacy implements common statistical operations such as mean (SD) with DP and can be directly integrated into DataSHIELD. More specifically, pure DP has been implemented, which means that the statistical noise added to the result is drawn from a Laplacian distribution. Privacy is therefore defined with a single parameter “ε” (which corresponds to the inverse of the noise variance) and it is called the privacy budget. The privacy budget ε controls the amount of noise injected in the computation and hence the privacy of the analysis, smaller the privacy budget ε, higher the noise, which means better privacy guarantees since the private information is better covered by the noise.

DP induces a trade-off between privacy and accuracy of the analysis [14] as privacy is ensured with noise, better privacy means a lower signal-to-noise ratio and less meaningful results. Conversely, when ε is high, the noise is reduced and privacy degrades. Choosing the right value for ε from a privacy standpoint is quite controversial [15] and is highly dependent on the context and the study considered.

dsPrivacy in the context of FA works by adding Laplacian noise locally at each center on the results computed on the local datasets before results are aggregated on a central node. This is referred to as local DP, as opposed to central DP, where the noise is added after the aggregation part on the central node. This paradigm is detailed in the Discussion section.

### Use Case: Differences in the Patient Drug Exposure of Patients With Non–Small Cell Lung Cancer Before and After the First Wave of the COVID-19 Pandemic

#### Context

In order to test the implementation of a federated platform with DP, a study was conducted using real-world oncology data, with the aim of analyzing the differences in the patient drug exposure of patients with non–small cell lung cancer before and after the first wave of the COVID-19 pandemic. Specifically, the analyses were based on the first line of treatment, including its duration and disease progression at 24 months. The study was carried out in 3 hospitals: Foch Hospital, Toulouse University Hospital Center, and Reims University Hospital Center.

#### Patient Selection

The inclusion criteria were adult patients with metastatic non–small cell lung cancer treated with chemotherapy, immunotherapy, antiangiogenic therapy, or any combination of these treatments at Foch, Toulouse, and Reims centers between March 2019 and March 2021. The list of considered treatments is available in Multimedia Appendix 1.

The exclusion criteria included patients objecting to the reuse of their data, protected adult patients (patients under curatorship, tutorship, or advisership), patients undergoing a clinical trial, patients whose follow-up did not start in one of these centers, patients who are not immediately metastatic, and patients with an epidermal growth factor receptor mutation, anaplastic lymphoma kinase translocation, or ROS proto-oncogene 1 mutation.

For this study, the objective was to have 25 patients per period and per site (ie, 50 patients per center). Initially, patients were preselected using the inclusion criteria present in the chemotherapy prescription software CHIMIO, namely the date and type of treatment. Since the other criteria for inclusion and exclusion were not available in CHIMIO, physicians in each center manually assessed whether the patients could be included in the study.

#### Variables

In this study, the variables of interest were the duration of the first line of treatment and the disease progression at 24 months following or during the first line of treatment. The start of a second line of treatment or death following (before the start of a second line) or during the first line of treatment were the proxies used for disease progression at 24 months. These events had to have occurred during the observation period, which was 2 years after the patient’s inclusion in the study. The explanatory variable was the patient’s inclusion period, before the first wave of COVID-19 (March 1, 2019, to March 1, 2020) or after the first wave of COVID-19 (March 2, 2020, to March 31, 2021). Some potential confounders were also studied, such as age, gender, BMI, blood creatinine level, and type of treatment. All these variables were extracted from the CHIMIO software and are listed in Multimedia Appendix 2.

#### Data Harmonization

In this study, a physician visited each participating site to ensure consistent variable definitions, coding standards, and data formats. We chose Fast Healthcare Interoperability Resources (FHIR) as the initial data standard [16,17], due to its maturity and support in oncology, and the data model is available in Multimedia Appendix 3. Harmonization involved reviewing data dictionaries at each hospital, resolving semantic ambiguities (eg, different coding for treatments), and ensuring uniform data types and units. This manual process, though time-consuming, proved crucial for producing a common dataset compatible with federated queries. In future implementations, automated or semiautomated harmonization workflows, potentially protected by DP to further safeguard patient privacy during preprocessing, could streamline this step. To facilitate the analyses, a tabularized version of the FHIR standard has been produced and the correspondence between the variables of interest used in the datasets and the FHIR attributes are presented in Multimedia Appendix 2.

#### Statistical Definition Without Differential Privacy

First, univariate and bivariate descriptive statistics were performed for the variables of interest, the explanatory variables and the confounding factors. For the qualitative variables, percentages per category were calculated. For the quantitative variables, the mean but also the 5th, 25th, 50th, 75th, and 95th percentiles were computed. The minimums and maximums were not computed because these values are considered disclosive (eg, the presence of an outlier) in DataSHIELD standard configuration.

Then, to ensure the absence of confounding factors, covariates for which there was a difference between the 2 groups (before and after the first COVID-19 wave) in bivariate analysis with a *P* value of <.20 were included in the multivariate models. A Student *t* test was used for quantitative variables. A chi-square test was used for qualitative variables with at least 5 elements in each class. Otherwise, a Fisher test was used.

Finally, to investigate a difference in management between patients in the first and second waves, a linear regression was performed. The variable of interest was the duration of first-line treatment at the metastatic stage and the explanatory variable was the period (before or after the first wave). The duration of treatment was defined as the period from day 1 to the last day of the first line. A multivariate survival analysis (Cox model) was also performed in which the event of interest was the disease progression at 24 months. The explanatory variable was also the period (before or after the first wave). Potential confounders identified in the previous step were included in both multivariate analyses. A difference was considered significant if the CI did not contain 1 for linear regression and survival analysis.

#### Statistical Definition With Differential Privacy

The univariate and bivariate statistical analyses presented in above were also performed with DP. The global strategy used to implement DP has been discussed in “Use of Differential Privacy” section and some details for each part of the study are detailed here.

All the univariate analyses run code from PyDP [11] that we wrapped in dsPrivacy. As adding DP preserves privacy, the minimum and maximum values could be computed instead of 5th and 95th percentiles. Then bivariate analyses were carried out. For quantitative variables, an implementation of the Student *t* test is used and from the result of this test, a *P* value is inferred. For qualitative variables, occurrence tables are computed (under the hood, the function tableDP from DPPack [18] is used in each center and all the tables are summed) and then a chi-square or Fisher test is performed with the R functions “chisq.test” and “fisher.test.” Linear regression and Cox models were not implemented with DP.

Determining appropriate ε values is challenging and context-dependent. In this study, our priority was to maintain analytical use. After testing lower ε values (closer to 1) that yielded excessive noise and unusable results, we selected *ε*=5.0 for univariate analyses and *ε*=60.0 for complex operations like the Student *t* tests. While *ε*=60.0 is higher than common DP standards in theoretical literature, this choice reflects our small sample size (~50 patients/center) and the need to ensure meaningful statistical inference. In scenarios with more patients or improved aggregation techniques (eg, secure aggregation enabling central DP), lower ε values could be achieved. As this was a feasibility study, we highlight that future work should explore more advanced DP mechanisms, larger sample sizes, and improved cryptographic methods to reduce the required privacy budget.

### Ethical Considerations

From an ethical and regulatory standpoint, this study complied with the MR-004 reference methodology and adhered to GDPR requirements since DP alone does not classify data as anonymized under current regulatory frameworks. However, the use of DP strengthens data protection, and aligns with emerging privacy regulations by minimizing data exposure risks. These regulatory environments emphasize data sovereignty, patient consent, and robust security, all of which are supported by our federated, privacy-enhancing architecture. All patients enrolled in this study were informed individually, and those who exercised their right to opt out were removed from the study. This is an observational study and the research ethics committees of all three centers in Toulouse, Reims, and Foch have confirmed that no ethical approval is required and have authorized this project on their patient data.

## Results

### Deployment of Federated Architecture in the Three Centers

**Figure 1.**
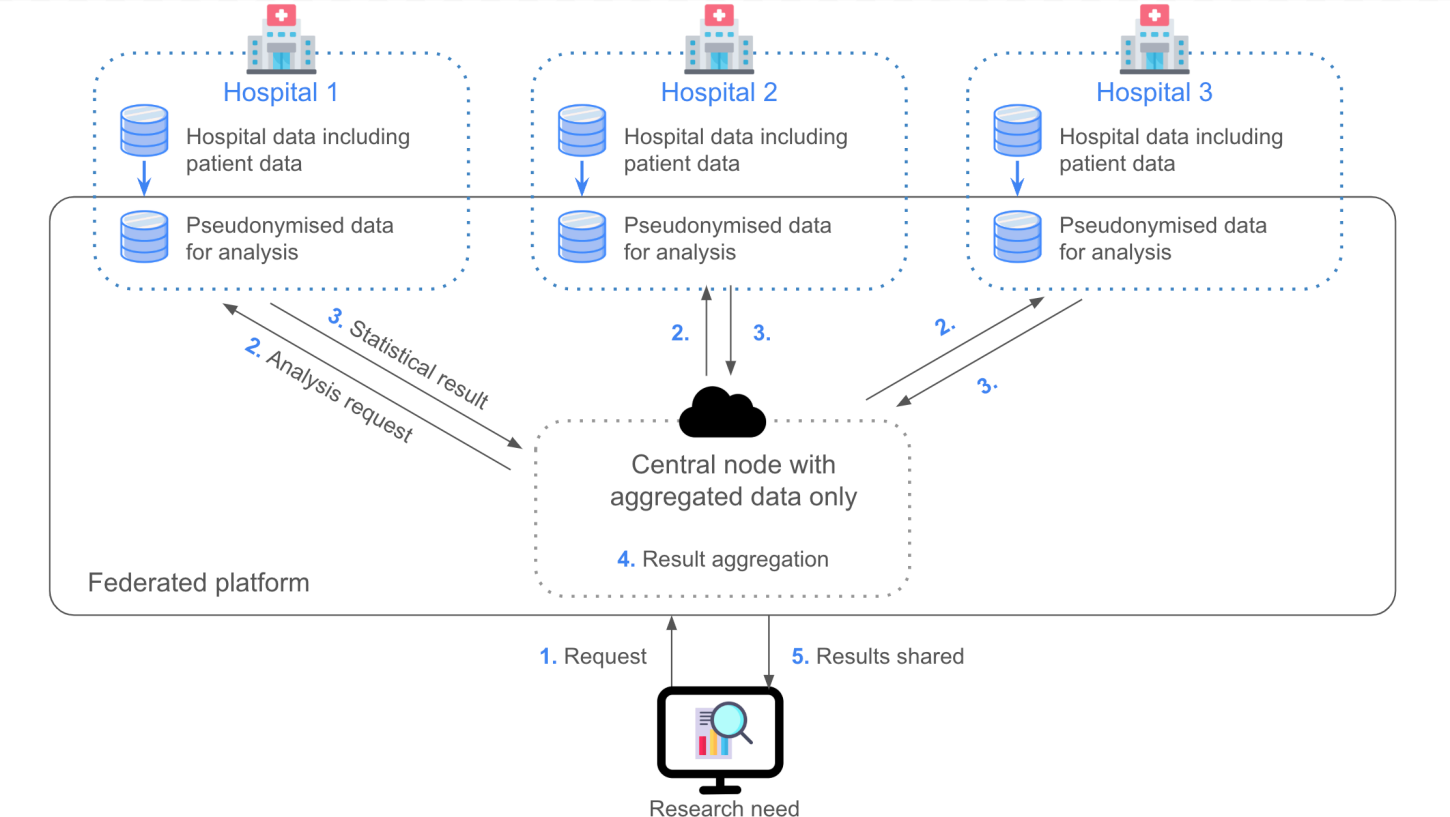
Federated architecture diagram.

The schema of our federated architecture is given in Figure 1. Researchers connect through a VPN to a central node hosted on a cloud provider–certified ISO (International Organization for Standardization) 27001 standard. This central node coordinates the execution of the queries on the remote data assets hosted in the different hospitals and aggregate all the responses received. Each hospital exposes through the private network pseudonymized datasets, which originate from their local harmonized data architecture, but which are completely isolated on a dedicated infrastructure for security purposes. Each hospital receives and executes remote requests from the central node that match local permissions set in DataSHIELD regarding data assets, authorized functions, and legitimate users. This ensures that hospitals keep full sovereignty on how their data are exploited.

One key element is deploying a secure private network between all stakeholders, ensuring that each hospital only exposes pseudonymized data within a controlled environment. Although these pseudonymized datasets were not themselves protected by DP before analysis (since DP is applied at query-time), the secure network, encryption (TLS), and isolation measures minimize risks. We did not use “Let’s Encrypt” for certificates due to internal security policies favoring certificates from recognized authorities and the controlled VPN environment lacking public domain validation. Instead, a custom approach using a trusted certificate authority provided both strong authentication and encryption tailored to our network configuration.

### Flowchart

**Figure 2.**
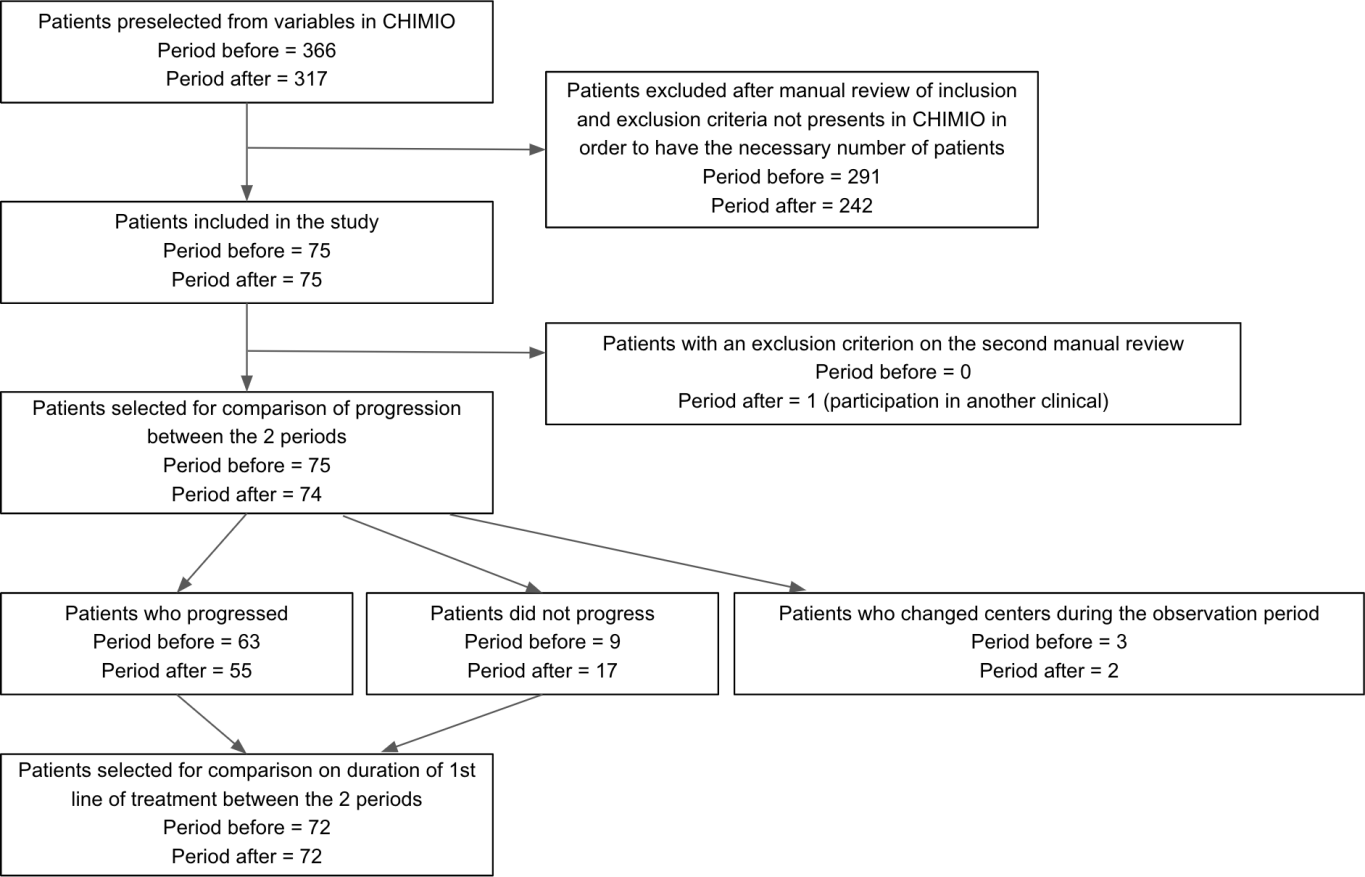
Patient selection and exclusion process between two periods: before and after the first wave (BW and AW).

The flowchart is presented in Figure 2. In total, 75 and 74 patients from respectively before and after the first wave were included in the analysis comparing disease progression. Of the patients in the period before, 63 had progressed, 9 were progression-free, and 3 had changed groups during the observation period and were therefore censored at the time of transfer. Of the patients in the period after, 55 had progressed, 17 were progression-free and 2 had changed groups during the observation period and were therefore censored at the time of transfer. Thus, after exclusion of patients who changed centers, 72 patients from each period were included in the analysis of the duration of the first line of treatment.

### Bivariate Analysis and Selection of Potential Confounders

Combined univariate analyses without and those with DP are presented in Multimedia Appendix 4. Combined bivariate analyses without and with DP are presented in Tables1  2, respectively. Direct comparison between DP and no-DP analyses across all periods is available in Table 3. In analysis without and with DP, the covariates selected for multivariate analyses were the treatment type (<.001) only.

**Table 1.** Bivariate analyses without differential privacy.

Variables	Patients (period before; n=75)	Patients (period after; n=74)	*P* value
Variables of interest			
Disease progression, n (%)			.21
No	12 (16)	19 (25.7)	
Yes	63 (84)	55 (74.3)	
Missing	0 (0)	0 (0)	
First line duration (days)			.09
Mean (SD)	179.7 (267.1)	255.2 (274.6)	
Median (IQR)	75.7 (14.7‐190)	141.8 (45.1‐420.8)	
5th and 95th percentiles	0‐592.4	15‐792.8	
Missing, n (%)	(0)	(0)	
Covariables			
Organization, n (%)			.99
Toulouse University Hospital	25 (33.3)	25 (33.8)	
Reims University Hospital	25 (33.3)	25 (33.8)	
Foch Hospital	25 (33.3)	24 (32.4)	
Missing	0 (0)	0 (0)	
Gender, n (%)			.79
Female	33 (44)	30 (40.5)	
Male	42 (56)	44 (59.5)	
Missing	0 (0)	0 (0)	
Age (years), n (%)			.38
<55	12 (16)	10 (13.5)	
55‐65	30 (40)	23 (31.1)	
>65	33 (44)	41 (55.4)	
Missing	0 (0)	0 (0)	
BMI (kg/m²), n (%)			.39
<18.5	13 (17.3)	19 (25.7)	
18.5‐25	34 (45.3)	27 (36.5)	
>25	28 (37.3)	28 (37.8)	
Missing	0 (0)	0 (0)	
Treatment category, n (%)			<.001
Chemotherapy	46 (61.3)	30 (40.5)	
Chemotherapy+angiogenesis inhibitor	6 (8)	0 (0)	
Chemotherapy+immunotherapy	7 (9.3)	33 (44.6)	
Immunotherapy	16 (21.3)	11 (14.9)	
Missing	0 (0)	0 (0)	
Creatinemia (µmol/l)			.47
Mean (SD)	66.7 (20.3)	64.3 (18.9)	
Median (IQR)	60.0 (54.7‐73.3)	63.7 (55.1‐73.1)	
5th and 95th percentiles	46.7‐98.8	41.1‐82.8	
Missing, n (%)	0 (0)	1 (1.4)	

**Table 2.** Bivariate analyses with differential privacy.

Variables	Patients (period before)	Patients (period after)	*P* value
Variables of interest			
Disease progression (*ε^a^*=5.0), n (%)		.27
No	12 (16)	18 (24.7)	
Yes	63 (84)	55 (75.3)
Firstline duration (days ; *ε*=5.0, except *P* value: *ε*=60.0)		.051
Mean (SD)	189.5 (231.1)	287.4 (286.3)	
Median	81.8	132.6
Range	0.3‐1359.5	8.5‐827.1
Covariables			
Gender (*ε*=5.0), n (%)		.92
Female	32 (43.2)	30 (41.1)	
Male	42 (56.6)	43 (58.9)
Age (years; *ε*=5.0), n (%)		.37
<55	12 (16.2)	10 (13.7)	
55‐65	29 (39.2)	22 (30.1)
>65	33 (44.6)	41 (56.2)
BMI (kg/m²; *ε*=5.0), n (%)		.38
<18.5	13 (17.1)	19 (25.7)	
18.5‐25	34 (44.7)	27 (36.5)
>25	29 (38.2)	28 (37.8)
Treatment category (*ε*=5.0), n (%)		.001
Chemotherapy	46 (61.3)	31 (39.7)	
Chemotherapy+angiogenesis inhibitor	6 (8)	0 (0)
Chemotherapy+immunotherapy	7 (9.3)	35 (44.9)
Immunotherapy	16 (21.3)	12 (15.4)
Creatinemia (µmol/L) (*ε*=5.0 except for *P* value: *ε*=60.0)		.53
Mean (SD)	65.4 (18.9)	61.9 (17.1)	
Median	64.4	65.4
Range	48.8‐88.9	37.4‐86.1

a *ε*: privacy budget.

**Table 3.** Comparison of bivariate analyses across all periods (before and after), with and without differential privacy.

Variables	Global results (before and after combined) without DP^a^	Global results (before and after combined) with DP (*ε^b^*=5.0)
Variables of interest		
Disease progression, n		
No	31	31
Yes	118	118
First-line duration (days)		
Mean (SD)	217.2 (269.4)	198.9 (286)
Median	113.5	126.4
Range	0.5‐785.6	0.1‐524.8
Covariables		
Gender, n (%)		
Female	63	63
Male	86	87
Age (years), n		
<55	22	21
55‐65	53	52
>65	74	75
BMI (kg/m²), n		
<18.5	32	32
18.5‐25	61	61
>25	56	56
Treatment category, n		
Chemotherapy	76	76
Chemotherapy+ angiogenesis inhibitor	6	6
Chemotherapy+ immunotherapy	40	41
Immunotherapy	27	27
Creatinemia (µmol/L)		
Mean (SD)	65.5 (19.6)	64.6 (15.15)
Median	62.3	64.3
Range	43.9‐92.8	45.6‐83.7

a DP: differential privacy.

b *ε*: privacy budget.

### Comparison of the Progression Between Two Periods (Cox Model)

In the analysis without DP, the hazard ratio of the cox model for the period before compared with the period after the first wave was 0.98 (95% CI 0.65-1.46). As the 95% CI includes 1, the two periods were not considered significantly different in terms of disease progression at 24 months after adjustment for a significant covariate (treatment type).

### Comparison of the Duration of the First Line of Treatment Between the Two Periods (Linear Regression)

In the analysis without DP, the coefficient of linear regression for the first period compared with the second one was –13.84 (95% CI –103.82 to 76.14). As the CI includes 1, the two periods were not considered significantly different in terms of first line duration after adjustment for significant covariate (treatment type).

## Discussion

### Principal Findings

In this study, a federated analysis with and without DP was performed on a real-world study in oncology. The real-world study showed consistent results in both settings. In particular, identical conclusions were drawn regarding the lack of difference in first-line treatment duration and disease progression at 24 months in non–small cell lung cancer patients treated before or after the first wave of COVID-19 pandemic.

Real-world deployment in 3 centers has shed light on some key aspects in terms of security. Regarding tools, DataSHIELD offers a lot of flexibility as it allows the use of custom libraries such as dsPrivacy implemented as part of this study. The downside is that community libraries are not always well maintained and bugs can be encountered (eg, dsStats had to be fixed manually). DataSHIELD builds upon Opal [19], a convenient data management application which comes with straightforward but very satisfactory user management and makes it easy to configure access to specific data to specific users. Opal is also secured by design and enforces many good security practices like CSRF (Cross-Site Request Forgery) and HTTPS usage. However, available docker images to deploy it have several major known security vulnerabilities and do not seem regularly updated. Regarding network configuration, setting up strong security standards across all sites has proved challenging. An IpSec bridge was set up to enable secure communication between the central node and the hospitals. Communication among all stakeholders was a key factor for this step and formal processes (network schema, requirements formalization, debugging methods, and so on) proved decisive in order to facilitate discussions. Regarding certificates to secure communications inside the private network, the primary intention was to use certificates signed by hospitals internal certificate authority because it would not match our security requirements so certificates signed by public certificate authority have been claimed. Some hospitals provided a Sertigo certificate but it was not natively recognized by the central node server. Another hospital used instead a public certificate generated by the central node manager (Arkhn). This last solution has proven to be the best solution in terms of efficiency and security, even if the domain names covered by these certificates are not representing the actual ownership of hosts.

Regarding data standards, we chose FHIR, since it appeared to be the most mature health standard in oncology at the beginning of this project [16,17]. However, Observational Medical Outcomes Partnership (OMOP) is developing extensions for oncology and is rapidly closing the gap [20,21]. As the FHIR standard corresponds to json files that are not very suitable for analytical purposes, we anticipate that moving to OMOP will be more convenient for future analyses.

Overall, our experience suggests that the most time and work intensive parts are building harmonized data models locally and setting up the network infrastructure. As both these steps are agnostic of the study considered, we anticipate that they can be easily leveraged to scale the number of studies carried out on this infrastructure.

On the DP side, it has been decided not to perform linear regression and Cox models with DP because implementing it with a moderate privacy budget was deemed too complex. Privacy budgets used in this study (especially for computing the *P* values) are quite high compared with the literature [15,22], in order to achieve reasonable accuracy on differentially private results but we have shown that we are able to derive similar results compared with plain FA. As underlined above, the appropriate level of privacy is highly dependent on the context considered. Here, given that this study was already compliant to the French MR-004 methodology without DP, DP was used by prioritizing usefulness over privacy. In other contexts, especially if DP becomes recognized as a legitimate and safe technology to process personal data, and hence benefits from a dedicated regulatory status, lower privacy budgets will probably be required (alongside privacy-focused pen tests). We have identified 3 directions to achieve this. The first direction is to increase the number of patients per center. This is the most straightforward option to improve the privacy vs accuracy trade-off, since the amount of noise to add to reach a certain privacy budget ε directly depends on the number of individuals. We have included close to 50 patients per center, which is quite low especially for some budget intensive operations for which we estimate that a thousand patients would be adapted. Second, by using central DP. If the noise is added on the central node, it is computed considering the sum of the patients across all the centers and enables lower noise for the same privacy budget. This is especially powerful if many centers are participating in the federated analysis. However, this means that by default the central node can see the contribution of each center without any noise which is a privacy breach if it cannot be trusted. A common solution is to implement secure aggregation [23], meaning that all contributions are hidden and are only disclosed after the aggregation operation, using cryptographic mechanisms such as secure multiparty computation [24], homomorphic encryption [25], or functional encryption [26]. Secure aggregation is not yet implemented in dsPrivacy and is left as future work. Finally, it can be achieved by improving the DP mechanism. We have used pure or ε-DP, which is a simple mechanism and which makes composition very simple, since the privacy budget of a sequence of operations is the sum of the budget of each operation. More complex mechanisms such as (ε, δ)-DP [6] or Rényi DP [27] provide more optimized composition properties [28] that allow for a tighter privacy budget management.

### Conclusions

The DARAH project illustrates FA are a practical method to conduct scientific projects while improving data privacy, by keeping patient data stored in the hospitals and leveraging their already existing data architecture. It highlights some key challenges to be anticipated and possible answers to ensure the success of this type of projects. It also shows that DP can be used in addition to FA to improve privacy guarantees, but more experimentation is needed to develop guidelines and best practices, especially around the trade-off between accuracy and privacy. Finally, in an emerging ecosystem where tools for FA and DP are not yet well integrated, the dsPrivacy library will prove useful for researchers who want to explore privacy-friendly analysis methods.

## Supplementary material

10.2196/59685 Multimedia Appendix 1 List of treatments of interest and their category.

10.2196/59685 Multimedia Appendix 2 Correspondence between the variables of interest used in the dataset and the FHIR (Fast Healthcare Interoperability Resources) attributes.

10.2196/59685 Multimedia Appendix 3 Data model to standardize data from centers in tabularized FHIR (Fast Healthcare Interoperability Resources) standard.

10.2196/59685 Multimedia Appendix 4 Combined univariate analyses across all centers without and with differential privacy.

## Abbreviations

CSRF: Cross-Site Request Forgery
DARAH: Distributed Analytics for Research in Hospitals
DataSHIELD: Data aggregation through anonymous summary-statistics from harmonized individual-level databases
DP: differential privacy
FA: federated analytics
FHIR: Fast Healthcare Interoperability Resources
ISO: International Organization for Standardization
OMOP: Observational Medical Outcomes Partnership
