# Supplementary material for: Federated Analysis With Differential Privacy in Oncology Research: Longitudinal Observational Study Across Hospital Data Warehouses
Source: JMIR Med Inform. 2025 Jul 31;13:e59685. doi: 10.2196/59685 (PMC12312987; doi:10.2196/59685)
Supplement: Multimedia Appendix 1 [file medinform-v13-e59685-s001.docx]

# **Multimedia Appendix 1: List of treatments of interest and their category**

| **Molecule** | **Category** |
| --- | --- |
| Pemetrexed | Chemotherapy |
| Paclitaxel | Chemotherapy |
| Pemcitabine | Chemotherapy |
| Vinorelbine | Chemotherapy |
| Docetaxel | Chemotherapy |
| Pembrolizumab | Immunotherapy |
| Atezolizumab | Immunotherapy |
| Nivolumab | Immunotherapy |
| Bevacizumab | Antiangiogenic |
| Carboplatine-Pemetrexed | Chemotherapy |
| Cisplatine-Pemetrexed | Chemotherapy |
| Cisplatine-Gemcitabine | Chemotherapy |
| Carboplatine-Paclitaxel | Chemotherapy |
| Carboplatine-Pemetrexed-Pembrolizumab | Chemotherapy+Immunotherapy |
| Carboplatine-Paclitaxel-Pembrolizumab | Chemotherapy+Immunotherapy |
| Carboplatine-Pemetrexed-Bevacizumab | Chemotherapy+Angiogenesis Inhibitor |
| Cisplatine-Pemetrexed-Bevacizumab | Chemotherapy+Angiogenesis Inhibitor |
