# Supplementary material for: Federated Analysis With Differential Privacy in Oncology Research: Longitudinal Observational Study Across Hospital Data Warehouses
Source: JMIR Med Inform. 2025 Jul 31;13:e59685. doi: 10.2196/59685 (PMC12312987; doi:10.2196/59685)
Supplement: Multimedia Appendix 2 [file medinform-v13-e59685-s002.docx]

**Appendix 2: Correspondence between the variables of interest used in the dataset and the FHIR attributes**

| **Variables of interest** | **Tabularized FHIR attributes** | **Additional information** |
| --- | --- | --- |
| **Patient Data at baseline** |  |  |
| Arm (BW vs AW) | ResearchSubject.actualArm | - |
| Inclusion Date | ResearchSubject.period.start | Correspond to the start of the 1st line of treatment |
| Exit Date | ResearchSubject.period.end | Correspond to the end of the 1st line of treatment |
| Year of birth | Patient.birthDate | Used to calculate the age at baseline  Range of value for age: 18-110 |
| Gender | Patient.gender | - |
| Weight | Observation.valueQuantity.valueDecimal | where Observation.code.coding.code = ‘29463-7’ (LOINC Code Body weight)  Range of value: 40-120  Used to calculate BMI |
| Height | Observation.valueQuantity.valueDecimal | where Observation.code.coding.code = ‘8302-2’ (LOINC Code Body height)  Range of value: 130-200  Used to calculate BMI |
| Creatinemia | Observation.valueQuantity.valueDecimal | where Observation.code.coding.code = ‘14682-9’ (LOINC Code Creatinine [Mol/vol] in Serum or Plasma)  Range of value: 10-400 |
| **Disease Data** |  |  |
| Disease Progression at 24 months  (evaluate by a switch to a second line) | Observation.valueQuantity.valueString | where Observation.code.coding.code = ‘97509-4’ (LOINC Code Cancer disease progression) |
| **Treatment Data** |  |  |
| Type | CarePlan.category[0].coding.code  CarePlan.category[1].coding.code | Used to evaluate the treatment type of the first line |
| Start of the 1st line of treatment | CarePlan.effectivePeriod.start | Used to calculate the duration of the first line |
| End of the 1st line of treatment  (Last administration) | CarePlan.effectivePeriod.end | Used to calculate the duration of the first line |

BMI: Body Mass Index
