# Supplementary figures and images for: Federated Analysis With Differential Privacy in Oncology Research: Longitudinal Observational Study Across Hospital Data Warehouses
Source: JMIR Med Inform. 2025 Jul 31;13:e59685. doi: 10.2196/59685 (PMC12312987; doi:10.2196/59685)

# **Appendix 3:** Data model to standardize data from centers in tabularized FHIR standard**
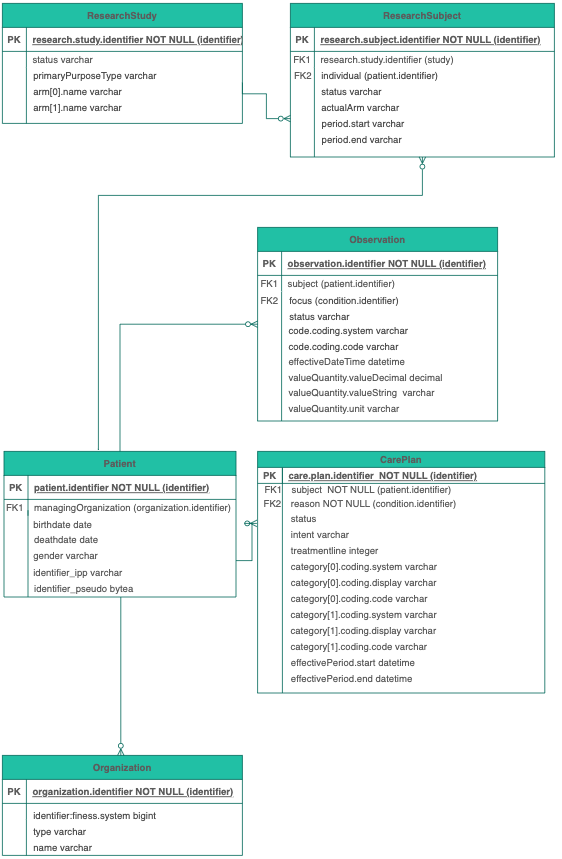
**

Supplement: Multimedia Appendix 3 [file medinform-v13-e59685-s003.docx]
