# Supplementary material for: Federated Analysis With Differential Privacy in Oncology Research: Longitudinal Observational Study Across Hospital Data Warehouses
Source: JMIR Med Inform. 2025 Jul 31;13:e59685. doi: 10.2196/59685 (PMC12312987; doi:10.2196/59685)
Supplement: Multimedia Appendix 4 [file medinform-v13-e59685-s004.docx]

# **Appendix 4: Combined univariate analyses across all centers without and with differential privacy (DP)**

| **Variables** | **Patients (n=149)**  **Without DP** | **Patients**  **With DP** |
| --- | --- | --- |
| **Explicative Variable** | | |
| Period, n(%) |  | ε = 5.0 |
| *BW* | 75 | 75 |
| *AW* | 74 | 76 |
| *Missing* | 0 | / |
| **Variables of interest** | | |
| Disease Progression, n(%) |  | ε = 5.0 |
| *No* | 31 | 31 |
| *Yes* | 118 | 118 |
| *Missing* | 0 | / |
| First Line Duration (days) |  | ε = 5.0 |
| *Mean (sd)* | 217.2 (269.4) | 198.9 (286.0) |
| *Median (Q25-Q75)* | 113.5 (35.4-289.0) | 126.4 |
| *Q5-Q95 / min-max* | 0.5-785.6 | 0.1-524.8 |
| *Missing, n(%)* | 0 (0.0) | / |
| **Covariables** | | |
| Organization, n(%) |  |  |
| *Toulouse University Hospital* | 50 | / |
| *Reims University Hospital* | 50 | / |
| *Foch Hospital* | 49 | / |
| *Missing* | 0 | / |
| Gender, n(%) |  | ε = 5.0 |
| *Female* | 63 | 63 |
| *Male* | 86 | 87 |
| *Missing* | 0 | / |
| Age, n(%) |  | ε = 5.0 |
| *<55* | 22 | 21 |
| *55-65* | 53 | 52 |
| *>65* | 74 | 75 |
| *Missing* | 0 | / |
| BMI, n(%) |  | ε = 5.0 |
| *<18.5* | 32 | 32 |
| *18.5-25* | 61 | 61 |
| *>25* | 56 | 56 |
| *Missing* | 0 | / |
| Treatment Category, n(%) |  | ε = 5.0 |
| *Chemotherapy* | 76 | 76 |
| *Chemotherapy+Angiogenesis Inhibitor* | 6 | 6 |
| *Chemotherapy+Immunotherapy* | 40 | 41 |
| *Immunotherapy* | 27 | 27 |
| *Missing* | 0 | / |
| Creatinemia (µmol/l) |  | ε = 2.0 |
| *Mean (sd)* | 65.5 (19.6) | 76.2 (70.8) |
| *Median (Q25-Q75)* | 62.3 | 62.1 |
| *Q5-Q95 / min-max* | 43.9-92.8 | 47.7-87.4 |
| *Missing, n(%)* | 1 (0.7) | / |

DP: Differential Privacy; BW: Before the wave; AW: After the wave; BMI: Body Mass Index; sd: Standard Deviation; ε: privacy budget
